# An Epitome of Braithwaite’s Retrospect

**Published:** 1860-05

**Authors:** 


ART. XII.—*An Epitome of Braithwaite's Retrospect of Practical Medicine and Surgery. In Five Parts.* By WALTER S. WELLS, M.D. Charles S. Evans: New York, 1860.

PARTS second and third of this valuable abridgment have been placed upon our table by Dr. Wells, who is at once author and proprietor. The labor of epitomizing so large a work as this must be immense, and we are sure the American editor deserves no little credit for the able and faithful manner in which he is executing his self-imposed task. Each number, as stated in our last issue, is furnished at one dollar, payable on delivery; a sum which must place the work within the reach of every member of the profession. We most earnestly commend Dr. Wells for his courage in issuing it at his own expense, and trust that he may not only not sustain any loss, but, on the contrary, find the enterprise adequately remunerative. Two more numbers will complete the analysis of the first forty volumes of the original work.

